# Genetic analysis of polymorphisms in the kalirin gene for association with age-at-onset in European Huntington disease patients

**DOI:** 10.1186/1471-2350-13-48

**Published:** 2012-06-21

**Authors:** Yu-Chun Tsai, Silke Metzger, Olaf Riess, Anne S Soehn, Huu Phuc Nguyen

**Affiliations:** 1 Department of Medical Genetics, University of Tuebingen, Calwerstr 7, Tuebingen, 72076, Germany

## Abstract

**Background:**

Huntington disease (HD) is caused by an expanded CAG repeat in the *HD* gene. Although the length of the CAG repeat strongly correlates with the age-at-onset (AAO), AAO in HD individuals may differ dramatically in spite of similar expanded CAG repeat lengths. Additional genetic or environmental factors are thought to influence the disease onset. Several modifier genes have been discovered so far but they do not fully explain the variability of AAO in HD. To potentially identify a novel genetic modifier, we analyzed single nucleotide polymorphisms (SNPs) in the *kalirin* (*KALRN*) gene. Kalirin is a protein crucially involved in spine plasticity and its interaction with huntingtin-associated protein-1 (HAP-1) and a potential protein dysfunction might contribute to spine pathogenesis in HD.

**Methods:**

The selected SNPs were genotyped by polymerase chain reaction-restriction fragment length polymorphism (PCR-RFLP) and association of SNPs with AAO was investigated with the framework of linear models in an analysis of variance and covariance.

**Results:**

Eleven SNPs in the *kalirin* gene were examined in an association study in European HD patients. The ten coding SNPs under investigation were monomorphic, whereas SNP rs10934657 in the promoter region showed a minor allele frequency >1%. An analysis of covariance together with the influence of the expanded *HD* allele was applied in 680 HD patients. SNP rs10934657 did not affect the AAO of the examined HD population.

**Conclusions:**

The results did not reveal an association between the analyzed *kalirin* polymorphisms and the AAO in HD. However, it does not exclude other SNPs of the *kalirin* gene as susceptible genetic modifiers.

## Background

Huntington disease (HD) is one of the most common autosomal-dominant neurodegenerative disorders caused by an expansion of an instable CAG repeat in the *HD* gene resulting in a polyglutamine tract near the amino terminus of the huntingtin protein (htt) [1]. The mutation leads to the selective loss of vulnerable neurons, notably medium spiny neurons in the caudate nucleus, which results in motor and cognitive impairment, personality changes and psychiatric illness [2]. The number of CAG repeats in the *HD* gene is the primary determinant of disease onset, however it only accounts for approximately 42-73% of the variance in age-at-onset (AAO) in HD [3,4]. Similar to several other neurodegenerative disorders, the detrimental gene alone does not fully determine the AAO in the course of the disease. Environmental factors, although not specifically defined yet, may contribute to different disease manifestation. The remaining variation of AAO may be due to modifier genes and seems to be strongly heritable [5]. Several genetic modifiers of HD have been identified so far, including huntingtin-associated protein-1 (HAP1) [6], ubiquitin C-terminal hydrolase 1 (UCHL1) [7,8], GluR6 subunit of kainate receptor (GRIK2) [9], the adenosinergic A2A receptor (ADORA2A)[10,11], autophagy-related protein 7 (Atg7) [12] and the peroxisome proliferator-activated receptor-γ coactivator 1α (PGC-1α) [13-15]. A recent review on HD modifiers was provided by Arning and Epplen [16]. These modifiers with their respective functions contribute to different aspects of pathogenesis in HD.

Cell death in the striatum and aggregation of the mutant huntingtin protein are pathological hallmarks of HD [17]. Morphological alterations of dendrites and spines are also found in HD patients and animal models [18]. Ferrante *et al*. showed truncated dendritic arbors, focal swellings on dendrites and spine loss in patients with a severe grade of HD [19]. Similar dendritic degeneration has been observed in R6/2 mice expressing mutant *huntingtin* exon 1 with 150 CAG repeats and transgenic mice expressing full length *huntingtin* cDNA [20,21]. Spine morphogenesis and plasticity are modulated by actin dynamics, which is regulated by Rho-like small GTPases (Rac, RhoA and Cdc42) and their GDP/GTP exchange factors (GEFs) [22]. Kalirin is a brain-specific, multifunctional Rho GEF encoded by the *KALRN* gene on chromosome 3q21.2 that generates several isoforms by alternative splicing [23]. Rat *kalirin-7*, corresponding to isoform 2 of the human *kalirin* gene, is undetectable at birth and increases during synaptogenesis [23,24]. It contains a SEC14 domain, a spectrin-like domain, a RhoGEF domain and a Pleckstrin homology domain (PH) [25] which controls multiple functions of the protein. Kalirin-7 activates Rac1 and regulates dendritic spine morphogenesis, plasticity and development [26,27]. Another probable link between *kalirin* and HD pathogenesis is huntingtin-associated protein-1 (HAP-1), a HD genetic modifier which interacts with the kalirin protein [6,28].

Although several susceptibility modifier genes for HD have been identified, they are not sufficient to explain the rest of the variance in AAO in HD. The goal of the present study was to investigate if SNPs in the *kalirin* gene also have a modifier effect on the AAO in HD. We specifically focused on the isoform-2 of the *kalirin* gene since it is the major splice variant in the adult brain, which integrates various signaling inputs and modulates dendritic spine maturation, plasticity and dynamics. In this regard, we analyzed one SNP in the promoter region and ten non-synonymous SNPs (D451E, Q520R, Q585E, G654W, T727S, R837Q, X1112E, D1326E, N1389H, E1588G) in the translated region of isoform-2 of the *kalirin* gene.

## Methods

### Subjects

A total of 680 unrelated European HD patients were analyzed. Among them, 320 patients were of German descent and 171 patients were of Italian descent. The remaining 189 patients were from other European countries [6,12,15]. AAO was determined by neurologists specialized in HD, usually as the time point when motor symptoms were first noticed. The mean AAO was 44.0 years (SD 13.0, age range 5–80 years). CAG repeat lengths in the *HD* gene had been tested in all patients and CAG numbers had been standardized in a reference laboratory. The number of the expanded CAG repeats ranged from 39 to 90. All healthy individuals are samples of the Centre d’Etude du Polymorphisme Human cohort (CEPH).

### Ethics

All participating individuals gave informed consent according to the Declaration of Helsinki. An ethics proposal was approved by the ethical review committee of the Medical Department of the University of Tuebingen (39/2003).

### Genotyping

The selected SNPs were genotyped by polymerase chain reaction-restriction fragment length polymorphism (PCR-RFLP). The target sequences were amplified with mismatch forward or reverse primers and digested with specific restriction enzymes. The mismatch primers were generated using dCAPs Finder 2.0 software (http://helix.wustl.edu/dcaps/dcaps.html) and optimized by Primer 3 program (http://frodo.wi.mit.edu/primer3/). Primer sequences for PCR amplification are shown in Table 1. PCR was performed in a final volume of 25 μl using 5 μl DNA, 1 μl of each primer, 1x buffer, 0.2 mM dNTP and 1.5U DNA Taq polymerase (BioTherm™). The cycling profile was as follows: 95°C 5′; [95°C 30″; respective annealing temperature for each SNP is indicated in Table 1, 30″; 72°C 1′] for 35 cycles; 72°C 5′ and stored at 10°C. Five μl of the PCR product were electrophoresed on 2% agarose gels and only samples with positive signals were used, yielding the total of 680 HD samples. The PCR products were incubated with 3U *Alu*I (rs10934657, rs111472457, rs61746078, rs2289838, rs2289838), 2.5U *Btg*I (rs35057827), 1.9U *Msc*I (rs13074913), 3U *BamH*I (rs61745397), 3U *Spe*I (rs112304715), 3U *Nde*I (rs2289838) or 3U *Sac*I (rs1062749) according to the manufacturer’s instructions (New England Biolabs, Inc., Beverly, MA, USA).

**Table 1:** Primer design for fragment length analysis

**SNP**	**Sequence (5′ → 3′)**	**PCR product (bp)**	**Annealing temperature (°C)**	**Restriction enzyme**
rs10934657	TGGCAAGAGGGAGAGG***A***G	139	55.2	*Alu*I
	CTTCCTCCTCTGTAAACCAGAGAGA			
rs111472457	CATCCGAGATGCAAGACCTAGA	156	58.5	*Alu*I
	CCGTGAGGGATTCGGAGT			
rs35057827	GCATGAGGTGTTACATCACCAGC***C***AC	123	60.9	*Btg*I
	CAATCCAGTCCAACACCTGCT			
rs61746078	CAGCAGGATGTACAGCAGGT	150	61.2	*Alu*I
	GTACGTATTCTGAGCCAC***AG***			
rs13074913	TCTACAAGGCAGCTCGACAC	136	58	*Msc*I
	AGGTCTTCCATCCATG***G***CC			
rs61745397	GCCAGGGACTCGGCTG***GA***	154	60.6	*BamH*I
	TCACCTCGATGGTGTACTGC			
rs112304715	CAGCAGGGACAGGATCTGCAC***T***	170	62.5	*Spe*I
	AGCCGCTTATGAGTCTGCTCT			
rs77832285	TCCTGAGTGAGCTCCTGCA***T***A***T***	110	59	*Nde*I
	GCTCGAACACCACATATTGC			
rs2289838	AGCCCGGAAGAAAGAATTTA	168	58.2	*Alu*I
	TGGATGTTGCCAAAGATGATA***A***G			
rs74389479	AAGTACGAGCAACTGCCTGAG	138	59.5	*Alu*I
	ATCAAAGAAGGTGCCCGCA***A***			
rs1062749	CTGCAAATTCGCCTTGTGGT	162	55.2	*Sac*I
	GCTGAAGTGGCTCCT***T***TAGAGCT			

Mismatch positions are highlighted in italics.

### **Statistical analysis**

Statistical analyses were performed as in our previous studies [12,15]. To determine allele and genotype frequencies and Hardy-Weinberg distribution of the tested genotypes GENEPOP software version 4.0.10 (http://www.genepop.curtin.edu.au/) was used. With the framework of linear models in an analysis of variance and covariance (JMP® Version 8.0.2 SAS institute, Inc., Cory, NC, USA) we investigated the modifying role of the polymorphic SNP rs10934657 in the *kalirin* gene on the AAO of HD. First, we applied a model of analysis of covariance with rs10934657 and the expanded *HD* allele as independent variables and the AAO as a dependent variable. The goodness-of-fit was assessed by the proportion of variation in the AAO that is explained by the coefficient of determination (R²). We obtained the best fit of our data and a minimization of the residuals by logarithmic transformation of the AAO and the CAG repeat number in the *HD* gene. To determine the effect of SNP rs10934657 on AAO by an analysis of variance and covariance, the effect of the expanded *HD* allele (HD CAG) was calculated alone, as well as with SNP rs10934657. When factor rs10934657 is added to the effect of the expanded *HD* allele (ΔR²), a change of R² would indicate a relative improvement of the model. This method would identify the percentage of the variance that is attributable to the candidate modifier loci when there is a significant P-value (P≤0.05).

## Results

**Figure 1:**
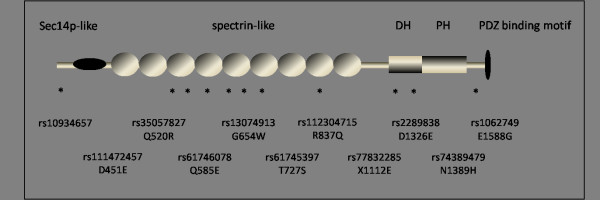
**Domain structure of the kalirin gene, isoform 2.** Dbl-homology (DH) and pleckstrin homology (PH) domains are responsible for the GEF activity of kalirin. The C terminus contains a unique 20 amino acid sequence with a PDZ domain-binding motif (STYV) that is specific for isoform 2. The localization of all SNPs examined in this study and rs numbers and changes on the protein level are indicated.

To explore the potential modifying effects of the *kalirin* gene on the AAO in HD, we chose eleven single nucleotide polymorphisms (SNPs) that were published in the NCBI SNP database at the start of this study. According to our hypothesis, via interaction with HAP-1 or due to protein dysfunction, the abnormal kalirin protein may contribute to spine pathogenesis in HD. Therefore, in this study we focused on SNPs that could potentially influence protein function or expression based on their positions in functional domains or regulatory regions. Accordingly, ten of the selected SNPs are non-synonymous and are located in spectrin-like domains (rs111472457 in exon 8, rs35057827 in exon 9, rs61746078 in exon 10, rs13074913 in exon 11, rs61745397 in exon 13, rs112304715 in exon 14, rs77832285 in exon 20), the Rho GEF domain (rs2289838 in exon 25, rs74389479 in exon 27) and downstream of the pleckstrin homology domain (rs1062749 in exon 32), respectively (Figure 1). SNP rs10934657 is in the 5′ untranslated region (5′UTR), a predicted promoter region. Accession numbers and alleles of the analyzed SNPs are shown in Table 2. We first screened these SNPs in 60 control samples (CEPH) to monitor the allele frequencies of each polymorphism. SNP rs10934657 in the 5′UTR region (C>T) was polymorphic with a minor allele frequency≥1% in controls whereas the ten coding SNPs were monomorphic in our cohort (Table 2). Therefore, SNP rs10934657 was selected for further genotyping of HD patients.

**Table 2:** Overview of the SNPs studied

**SNP ID**	**Chromosome position chromosome 3**	**Alleles^a^*(1/2)***	**Gene location**	**SNP type**	**Protein level**	**Controls (CEPH)**		**HD patients**				
						**Allele frequency**		**Genotype frequency**			**Allele frequency**	
						***1***	***2***	***1-1***	***1-2***	***2-2***	***1***	***2***
rs10934657	123812836	C / T	5′-UTR	Non-coding		0.8	0.2	487	174	19	0.844	0.156
rs111472457	124048782	G / T	exon 8	non-synonymous	D451E	1	0	-	-	-	-	-
rs35057827	124053260	G / A	exon 9	non-synonymous	Q520R	1	0	-	-	-	-	-
rs61746078	124066099	G / C	exon 10	non-synonymous	Q585E	1	0	-	-	-	-	-
rs13074913	124113985	T / G	exon 11	non-synonymous	G654W	1	0	-	-	-	-	-
rs61745397	124117557	T / A	exon 13	non-synonymous	T727S	1	0	-	-	-	-	-
rs112304715	124132486	A / G	exon 14	non-synonymous	R837Q	1	0	-	-	-	-	-
rs77832285	124165034	G / T	exon 20	non-synonymous	X1112E	1	0	-	-	-	-	-
rs2289838	124181433	G / T	exon 25	non-synonymous	D1326E	1	0	-	-	-	-	-
rs74389479	124196161	C / A	exon 27	non-synonymous	N1389H	1	0	-	-	-	-	-
rs1062749	124211666	G / A	exon 32	non-synonymous	E1588G	1	0	-	-	-	-	-

Chromosome positions were obtained from NCBI single nucleotide polymorphism (SNP) browser (http://www.ncbi.nlm.nih.gov/SNP/).

Reference sequence for coding SNPs: NM_003947.4.

The studied genotype distributions were consistent with Hardy-Weinberg distribution P=0.4716.

^a^ Alleles are described as 1 (wild type allele) or 2 (variant allele).

To examine the effect of the polymorphic SNP rs10934657 on disease onset, the respective genotypes were determined in a total of 680 HD patients. Genotyped allele frequencies are listed in Table 2. The allele frequencies of SNP rs10934657 in our European population were consistent with the HapMap-CEU population studies reported in the International HapMap project (http://www.hapmap.org) (C: 0.8; T: 0.2). In order to identify a possible modifying effect of SNP rs10934657 on the AAO of the analysed HD patients, an analysis of covariance together with the influence of the expanded *HD* allele was applied. Analysing the effect of the expanded CAG repeat in the *HD* gene itself, R² in the statistical model reaches a value of 0.5394 (Table 3). This indicates that the expanded *HD* alleles accounts for about 53% of the variance in the AAO, which is in good accordance to other studies [3,4]. However, the inclusion of SNP rs10934657 as covariant did not improve our model. This SNP did therefore not affect the AAO of the disease in the examined HD population (P=0.9713). Furthermore, there is no significant effect of the non-expanded CAG repeat on the AAO in our analysed population (data not shown).

**Table 3:** Effect of SNP rs10934657 on AAO in HD (Analysis of covariance)

**Variable**	**R²**	**ΔR²**	**p-value**	**Least significant number of patients**
*HD* CAG	0.5394		<0.0001	7
*HD* CAG + SNP rs10934657	0.5394	0	0.9713	70065

## Discussion

To our knowledge, this is the first study that examines an association of the *kalirin* gene with the AAO in HD. Previous studies have established a connection between *kalirin* variants and susceptibility to schizophrenia, Alzheimer disease, adult attention deficit hyperactivity disorder (ADHD), coronary artery disease and ischemic stroke [29-34]. In the present study, we hypothesized that isoform 2 of the *kalirin* gene, corresponding to rat kalirin-7, the predominant *kalirin* isoform in adult brain, may also contribute as a novel genetic modifier for HD based on its role in spine plasticity and its interaction with HAP-1.

Although the length of the expanded CAG tract in the *HD* gene is the main determinant of the HD phenotype, the manifestation of the disease is also modified by other risks, such as environmental or genetic factors. To date, two strategies have been applied for identifying genetic modifiers, the genome-wide approach and the candidate gene approach. The genome-wide studies are based on genetic linkage to search for specific chromosome regions, which might be associated with an alteration of age at neurological onset, including the HD-MAPS project which identified 6q23-24 as an association region [34] and the Venezuela pedigrees study [35]. The identified genomic regions in both studies were relatively large and it is difficult to detect specific modifiers, which are now analyzed with a combination of densely spaced SNPs and copy number probes. On the other hand, association studies that investigate candidate genes that are speculated to be involved in HD pathogenesis provide a straight forward option to identify these modifiers, although they are not comprehensive. Recent studies have demonstrated several genetic modifiers related to various mechanisms implicated in HD pathology, such as metabolic impairment, transcription dysregulation, oxidative stress and excitotoxicity [6,8,36,37]. Among them, the polymorphism T441M of HAP-1 showed an 8-year delay in AAO due to a tighter interaction of HAP-1 with mutant huntingtin (htt) protein and thus ameliorated htt-mediated toxicity [6]. HAP-1 is associated with huntingtin, dynactin p150/kinesin light chain (KLC), endosomal organelles and BDNF, suggesting its role in intracellular trafficking and endocytosis.

Here we examined polymorphisms in the *kalirin* gene due to its crucial role in spine plasticity and its interaction with HAP-1. Recently, several genome-wide association studies (GWAS) had revealed genetic associations of the *kalirin* gene with several diseases. An intronic SNP rs9289231 was associated with early onset coronary artery disease in an American white population [29], while intronic SNPs rs11712039, rs17286604 and rs11712619 were associated with ischemic stroke in a small Portuguese population [33]. A recent GWAS in a Japanese population showed that a missense mutation in the *kalirin* gene, P2255T (ss250607859), may be a genetic risk factor for schizophrenia [32]. In the present study we did not include any intronic SNPs, as we drew our attention to potential functional changes on the protein level. Also SNP ss250607859 was excluded from our analyses, as it affects another isoform of the *kalirin* gene.

## Conclusions

Among the eleven SNPs that were screened in our study, we found only SNP rs10934657 to be polymorphic in our European control cohort. However, in an analysis of covariance, there was no significant effect (P=0.9713) of this SNP on the AAO in our cohort of European HD patients. Also testing for additional factors, such as sex or German or Italian ancestry, did not reveal a significant effect either (data not shown). Further power analysis was performed to determine the population size that would be required to show potentially significant effects of rs10934657. Our calculations revealed a minimum of 70065 samples, indicating that this SNP is very unlikely to have a big impact on AAO in HD.

In summary, although the genetic variations of the *kalirin* gene investigated here showed no effect on the AAO in HD, this does not exclude other SNPs of the *kalirin* gene as susceptible genetic modifiers.

## Competing interests

The authors declare that they have no competing interests.

## Authors’ contributions

YCT carried out the molecular genetic analyses, participated in the statistical analysis of the data and drafted the manuscript. SM performed the statistical analysis. OR participated in the study design and reviewed the manuscript. AS and HPN conceived, designed and coordinated the study, interpretated the data and participated in writing of the manuscript. All authors read and approved the final manuscript.

## Pre-publication history

The pre-publication history for this paper can be accessed here:

http://www.biomedcentral.com/1471-2350/13/48/prepub
